# Redefining Success in Implant Dentistry: Beyond Mere Implant Placement

**DOI:** 10.1111/jre.70009

**Published:** 2025-07-03

**Authors:** Marcel F. Kunrath, Christer Dahlin

**Affiliations:** ^1^ Department of Biomaterials Institute of Clinical Sciences, Sahlgrenska Academy, University of Gothenburg Gothenburg Sweden; ^2^ Department of Dentistry School of Health and Life Sciences, Pontifical Catholic University of Rio Grande do Sul Porto Alegre Brazil

## 1 Background: Osseointegration and Implant Success (Past Century vs. Contemporary Status)

Since Prof. Branemark established the concept of osseointegration—“a direct structural and functional connection between bone and an implant”—[1]; implant dentistry has been revolutionized, progressing techniques and materials for rehabilitating patients who lost their teeth using dental implants, biomaterials, and implant‐supported prostheses [2]. After long years of clinical follow‐ups, the use of dental implants proved to be a reliable long‐term treatment for patients with unquestionable survival rates [2]. In the early ages of osseointegration, a successful osseointegrated implant was considered when after a healing period of 6–8 months, the bone‐anchored implant was completely stable and surrounded by healthy and non‐infected tissues, able to receive prosthetic loading and exercise mechanical function lasting long years in a patient [1]. After more than 50 years of successful use of dental implants, the clinical factors to declare a successful osseointegrated implant have changed [2, 3, 4, 5, 6]. Oral rehabilitation using dental implants has become a treatment dependent on specialized professionals who target handling complex long‐term cases combining surgical, periodontal, and prosthetic approaches, thinking beyond just implant insertion and early osseointegration. Currently, the concept of implant success and its long‐term osseointegration reveals a progressive complexity involving short and long‐term factors in implant dentistry. Biological, mechanical, functional, technological, genetic, prosthetic, esthetic, and health quality indicators (PROMs) can be cited as actual components to determine implant and osseointegration success [2, 3, 4, 5, 6]. Nevertheless, many of these factors may bring up miscellaneous opinions of what should be considered “implant success” or “osseointegration success” in the currently available knowledge, as well as, patients may show different feedback's about their satisfaction with implant treatments. Osseointegration success may be considered a stable implant in oral function with the absence of inflamed/contaminated surrounding tissues and peri‐implant bone loss. However, nowadays, the term “implant success” has achieved priority and embraces much more than healthy surrounding tissues; esthetics, correct implant positioning, treatment durability, changes in quality of life, and patient‐related outcomes are crucial in the contemporaneous definition [6]. The evaluation of solely peri‐implant tissue status is insufficient to achieve the current clinical scientific evidence needed to report quality of care, patient satisfaction, and treatment success in implant dentistry [6]. These multiple factors highlight the need for optimal surgical planning and safety protocols in implantology aiming for long‐term clinical durability. On the other hand, questionable treatments with dental implants and diverse clinical decisions may currently emerge regarding maintaining, re‐treating, or removing implants to redefine previous treatments or to achieve a different clinical scenario.

## 2 Contemporary Implant Dentistry: Immediate Resolutions, Implant Preservation, or Re‐Start Treatments?

Osseointegration discovery has surpassed more than half a century, and it is rare to find clinical cases in the literature reporting successful osseointegrated implants in function overpassing 30, 40, or 50 years of clinical follow‐ups [7]. Thus, some questions about long‐term osseointegration success arise:

1. Is it possible to achieve successfully osseointegrated implants lasting a complete life journey in the current concepts of implant dentistry success?
2. Are the current implant treatments focused on immediate resolution or long‐term clinical durability without replacement?
3. What will be the future long‐term success/survival rates of dental implants?

Nowadays, it is possible to notice a growing number of implants being explanted or re‐treated for different causes in dental clinics and/or institutions (e.g., incorrect implant‐positioning, esthetic issues, peri‐implantitis, bone loss, innovative prosthetic approaches, among others) [8]. Dental professionals have available an increased number of advanced technologies for implant treatments such as digital planning systems, innovative implant surfaces, guided and robotic surgeries, advanced biomaterials, and numerous prosthetic alternatives for bone‐anchored implant rehabilitation; allied with that, guidelines for implant treatment have been created and wide scientific evidence is freely available [2, 5, 9, 10]. Technologies and guidelines that should promote high implant survival and success rates, along with long‐term osseointegration without the need for additional interventions. However, the intense demand for esthetic treatments using dental implants, along with the desire for accelerated clinical resolutions, challenges the biological principle underlying osseointegration success and instigates many‐sided decisions regarding whether osseointegrated implants should be maintained or removed early to start new treatments [8, 11, 12]. An inspiring recent editorial [13] corroborates the last sentence; Prof. Lang mentioned that some negative emerging trends in implant dentistry, such as short‐term implant outcomes delivering fast financial returns, heavy marketing to sell and place implants, and/or guided surgeries performed without appropriate experience, may induce an increased number of questionable implant treatments. Nevertheless, implant dentistry should focus on implant treatments designed for long‐term success, and only cases where implant placement is clearly indicated and necessary should be undertaken [13].

Clinical guidelines for determining implant success in the current implantology may be complex to implement, as they involve multidisciplinary perspectives from dental professionals and diverse patient viewpoints. However, such guidelines are essential to increase the long‐life journey of osseointegrated implants and for establishing clear criteria when implants should be maintained or removed.

## 3 Future Perspectives: A New Proposed Definition of Success in Implant Dentistry

In contemporary implant dentistry, the term “implant osseointegration” should be understood as a complex and multi‐specialized biological process essential for achieving long‐term implant success and stability. Successful long‐term osseointegration maintenance in dental implants is dependent on an intricate union of pre‐, trans‐, and post‐operative elements to result in an outstanding clinical outcome, including steps such as pre‐operatory planning, general health of the patients, hard tissue adequacy, management of soft tissue, implant positioning, prosthetic rehabilitation, and long‐term and frequent maintenance follow‐ups [2, 3, 4, 5, 6, 7, 8, 9, 11, 12].

After implant‐loading (provisional or definitive), it is not adequate to evaluate implant success only by classical bone characteristics, as the osseointegrated implant is part of an interconnected system involving pieces interacting with soft tissues and exposed to the oral environment. The success of an implant should be evaluated using a multi‐specialized view, composed of clinician‐reported outcome measures (CROMs) and patient‐reported outcome measures (PROMs) [2, 3, 4, 5, 6, 7, 8, 9, 11, 12, 14]. Key elements such as peri‐implant bone stability, soft tissue stability surrounding the implant system, absence of peri‐implant disease/inflammation, appropriate prosthetic rehabilitation, crown esthetics (on the view of patient and clinician), soft tissue esthetics (on the view of patient and clinician), and last but not least, general patient satisfaction with the treatment, are essentials to define current implant success in long‐term implant treatments. Additionally to these elements, secondary factors indicators of treatment quality might interfere at the definition of a successful implant such as surgical/prosthetic planning, presence of keratinized soft tissue, vertical/horizontal measures of peri‐implant soft tissue, abutment design, crown design, implant maintenance, patient understanding of implant maintenance, and patient‐reported aspects (morbidity and quality of life) [5, 6, 9, 15]; these factors should be investigated with appropriate importance at the clinical stages if implant success was not diagnosed investigating the key elements cited on the Table 1.

**TABLE 1:** Key indicators for determining implant success.

Clinician‐reported outcome measures (CROMs)			
Indicators (domains)	Professional evaluations/observations	Assessment of implant success (rating)	Scientific evidence to support and consider in evaluating implant success for each indicator
Bone stability^a^ (radiographically observed)	The implant has not experienced bone loss beyond the initial remodeling (< 0.5 mm compared with the radiograph 0–1 year after loading, or bone level < 2 mm in the absence of baseline radiograph)	1—Yes 2—No	[6, 16, 17]
Soft tissue stability^a^	The soft tissue margin is at the same level of the contralateral element (esthetic zone only), and the soft tissue volume is satisfactory	1—Yes 2—No	[6, 12, 14, 15, 16, 18]
Inflammation^a^	The peri‐implant mucosa is not clinically inflamed or contaminated (absence of > 1 spot bleeding or profuse bleeding)	1—Yes 2—No	[6, 15, 18]
Prosthetic rehabilitation^a^	The prosthetic components did not experience technical complications or are exhibiting clinical complications (screw loosening, unbalanced occlusion, chipping, decementation)	1—Yes 2—No	[4, 6, 16, 17]

Patients‐reported outcome measures (PROMs)			
Indicators (domains)	Clinical evaluations/observations	Assessment of implant success (rating)	Scientific evidence to support and consider in evaluating implant success for each indicator
General satisfaction^a^	The patient is fully satisfied with the overall implant rehabilitation	1—Yes 2—Partially 3—No	[3, 6, 16, 19, 20]
Esthetics^a^	The patient is fully satisfied with the esthetics of the implant rehabilitation (color, crown, soft tissue margins, soft tissue texture)	1—Yes 2—Partially 3—No	[3, 6, 19]
Function^a^	The patient is fully satisfied with the masticatory function of the implant rehabilitation	1—Yes 2—Partially 3—No	[6, 16, 19, 20]

^a^ Score 1 for all the domains indicates implant success.

Therefore, a table (Table 1) describing current relevant elements that may define the success of an osseointegrated implant in function is proposed in this perspective article. These indicators may be divided into two important sections to evaluate a successful implant status: 1—clinician‐reported outcome measures (CROMs) and 2—patient‐reported outcome measures (PROMs). A combination of positive scores (1—Yes) for all mentioned indicators results in a suggested implant success. Failures in one or more of these indicators may determine the requirement of different levels of interventions/observations according to the level of inconsistency, such as surgical intervention, prosthetic modifications or replacement, frequent clinical maintenance, re‐treatments, and/or early implant removal. This suggested guide may assist in establishing novel studies and guidelines for diagnosing successful treatments with dental implants in contemporary implant dentistry.

Finally, this contemporary definition will help clinicians visualize the complexity of inserting dental implants aiming for long‐term implant success. Additionally, this report serves to raise awareness of the requirement to think beyond immediate resolutions in implant dentistry and instead target dental implant treatments for long‐term longevity.

## Disclosure

AI Statement: This manuscript did not use artificial intelligence in any capacity.

## Conflicts of Interest

The authors declare no conflicts of interest.

## Data Availability

The authors have nothing to report.
